# Does LSD Have Any Therapeutical Value In Mental Disorders? A Non-Systemic Review

**DOI:** 10.1192/j.eurpsy.2022.2272

**Published:** 2022-09-01

**Authors:** L. Silva, L. Bravo

**Affiliations:** Unidade Local de Saúde do Baixo Alentejo, Psychiatry, Beja, Portugal

**Keywords:** LSD, psychedelics, mental disorder, Cerebral Plasticity

## Abstract

**Introduction:**

Lysergic acid diethylamide (LSD) a semi synthetic hallucinogen, was first synthetized by Albert Hofmann in 1938 and its psychological properties were not discovered until 1943. There was a brief renaissance of its use in psychiatry, specifically in assisted psychotherapy for the treatment of alcoholism, neurosis and psychosomatic disorders. However, research with LSD and others psychedelics substances rapidly came to an end with its broad international prohibition in the 1970´s.

**Objectives:**

This work aims to provide an understanding on the potential benefits of LSD in mental disorders, as well as its mechanism of action and effects on human brain.

**Methods:**

A non-systematic review was performed on PubMed database, using the key words “LSD, mental disorder, neuroimaging, cerebral plasticity, psychedelics, substance use disorder”. Websites of the Berkley Foundation and Multidisciplinary Association For Psychedelic Studies (MAPS) were also searched.

**Results:**

Significant evidence has emerged that LSD may have a role in the treatment for some mental disorder, including drug and alcohol dependence and anxiety associated with life threatening diseases. Evidence based on modern neuroimaging technique suggest that LSD alters the brain blood flow, electrical activity, and network communication patterns. Preclinical evidence also demonstrated that psychedelics affect neuroplasticity after acute and chronic administration.

**Conclusions:**

Based on the available evidence LSD when administered safely in a methodologically supervised psychotherapeutic setting can have a potential use for certain psychiatric conditions, suggesting that larger controlled studies are warranted

**Disclosure:**

No significant relationships.

